# Informing care pathways and policies for children and youth with Indigenous perspectives to advance Canada's National Autism Strategy

**DOI:** 10.3389/fpsyt.2022.916256

**Published:** 2022-09-15

**Authors:** Celina Antony, Madison Campbell, Stephanie Côté, Grant Bruno, Carolyn Tinglin, Jonathan Lai

**Affiliations:** ^1^Department of Health Sciences, McMaster University, Hamilton, ON, Canada; ^2^Autism Alliance of Canada, Toronto, ON, Canada; ^3^Department of Pediatrics, University of Alberta, Edmonton, AB, Canada; ^4^Samson Cree Nation, Maskwacis, AB, Canada; ^5^Department of Education, Law, and Society, Simon Fraser University, Burnaby, BC, Canada; ^6^Institute of Health Policy Management and Evaluation, Dalla Lana School of Public Health, University of Toronto, Toronto, ON, Canada

**Keywords:** autism, policy, Indigenous, services, culturally safe care, Canada, BIPOC

## Abstract

In Canada, most services for Autistic people are provided by provincial and territorial governments. However, support for Indigenous Peoples (First Nations, Inuit, and Métis) are under federal responsibility and are outlined by a set of treaties and agreements with the Crown and a few regional governments. This patchwork results in barriers in service access and navigation challenges in many under-resourced communities, including under-diagnosis and potentially life-threatening outcomes. Designing equitable policy structures and processes would reduce harms and meaningfully interface with Indigenous and other racialized communities. The objective of this Policy Practice Review is to provide a framework for the discovery of appropriate care strategies addressing the conceptualization of autism in Indigenous Peoples and to understand the interactions between racialized Autistic peoples and the Criminal Justice System. First, we conducted environmental scans of publicly-accessible government services available in Canada pertaining to autism in Indigenous communities and the justice system, and explored the dissonance with beliefs and perceptions of autism in Northern Indigenous communities. Second, we focused on the interactions of Indigenous and other racialized populations, with an emphasis on Autistic children and youth with the justice system, an interaction that is often life-altering, downstream, and detrimental to health and wellbeing. The implications of this work include identifying the need for Indigenous-led knowledge and policy recommendations for Canada's upcoming National Autism Strategy, informing the need for culturally appropriate multidisciplinary care and facilitating the coordination between health and social services for these communities.

This Policy and Practice Review presents the systems and structures that challenge inclusive policy design and lead to barriers for Autistic people in health, social services, and justice. As part of the development of a National Autism Strategy (NAS) in Canada, there is the opportunity to acknowledge, recognize, and integrate the unique populations that are affected by the systems and structures in place. The current review explores issues encountered by Indigenous Peoples and other racialized groups with the concept of autism and recommendations for the upcoming Canadian strategy. The interactions examined were in relation to health, social services, and justice systems in Canada. When accessing the various programs and services in these systems, Indigenous Peoples in Canada are required to meet the government-established criteria for Indian status. The Canadian Constitution recognizes three distinct groups of Indigenous Peoples: First Nations, Métis, and Inuit (1). Indian status is governed by the Indian Act of 1876, a Canadian federal legislation that authorized the federal government to regulate the lives of First Nations Peoples registered under the Act. This legislation involves the political management of governance structures used within First Nations communities, rights to practicing and teaching Indigenous cultural traditions, and the regulation of Indigenous resources and lands in the form of reserves (2). Indian status is often required for eligibility to varying federal or territorial government benefits, services, and programs (3). The Indian Act resulted in many jurisdictional challenges, a well-known challenge being access to healthcare. Whereas, health services are under the responsibility of Canada's 13 provinces and territories, funding and delivery of health services to Indigenous Peoples are the responsibility of the federal government according to the Indian Act (4). The current structure continues to cultivate mistrust among Indigenous Peoples with regards to existing healthcare services, and this is especially prevalent for developmental disabilities such as autism (5).

With Canada's current work to develop an NAS that adequately serves the needs of Autistic people in Canada, the systematic barriers and inequities that affect racialized communities require critical reflection in the development of appropriate and culturally safe services. Although there are more than 630 First Nations communities situated across Canada, there continues to be an underrepresentation of Indigenous Peoples diagnosed with autism in reports of autism prevalence (6, 7). For instance, a prevalence study found significant under-representation of autism among Indigenous children in Manitoba and Prince Edward Island (8). According to Lindblom, this may stem from the lack of culturally safe diagnostic services and research available within these communities (7). Research has shown that engagement in traditional cultural practices can help to reduce personal challenges and improve social development in Autistic First Nations children (7). Hence, there are evident cultural influences affecting the unique experiences of Autistic Indigenous Peoples, which require additional focus in research to develop culturally appropriate and safe services for Indigenous Peoples.

Community members who use traditional knowledge and healing practices often experience stronger Indigenous identity and improved spiritual health compared to those who did not use traditional methods (9). Despite the value that traditional practices hold for Indigenous Peoples, the diagnostic supports and services available within Indigenous communities often take a Western approach to screening and detection. This dissonance between Indigenous traditional values and the services available for Autistic Indigenous Peoples emphasizes the need for policy and practice which critically consider the historical contexts that shape the interactions of racialized peoples with social systems, such as health, school, and the CJS.

## The unique context in which Indigenous and Black Autistic communities exist and interact within Canada

Indigenous Peoples in Canada have faced a prolonged history of colonialism and systemic oppression that have affected their interactions in seeking health and social services. This has taken several forms in addition to the Indian Act, which include but are not limited to: the Indian Residential School system, a system separating Indigenous Peoples from their cultural identities and traditions, assimilating Indigenous students to colonialist societies; the sixties scoop, the mass removal of Indigenous children from their families in the 1960s into the child welfare system; the continual neglect of issues depriving Indigenous communities of basic living needs, such as access to clean water supply on reserves; as well as the implementation of Indian hospitals during the tuberculosis epidemic, which advanced the notion that Indigenous Peoples posed a potential threat to non-Indigenous populations (10–12). These experiences, among countless others, have left a deep imprint in Indigenous cultural conceptions of health and illness, and continue to propagate the mistrust felt by Indigenous Peoples in the Western-oriented health and social systems. Acknowledging the experiences of racialized Autistic individuals who have been forced to participate in Western defined services such as health care and justice systems would be a step toward building trust, and reducing the continued structural violence that shapes Canada's current health programs and services.

Along with a clear disconnect between Western and Indigenous approaches to mental health and wellbeing, the Western approaches adopted within government-funded programs embed in a history of colonialism, institutional racism, systemic barriers, and inequitable policies and practices that continue to affect Indigenous communities (13, 14). To our knowledge, wellness within Indigenous communities can only be adequately evaluated using a holistic model, which seeks balance between all physical, emotional, social, and spiritual aspects of life (13). Specifically, supports and services available to Indigenous communities do not address Indigenous Ways of Knowing, the process of observing, discussing, and making sense of new information traditionally used among Indigenous Peoples (14, 15). This misalignment prevents access to meaningful supports for Indigenous Peoples. For instance, under Canada's Jordan's principle, Indigenous children and youth with autism have the right to care whether located on or off-reserve; however, this principle does not consider youth transitioning into adulthood that may still require funding to access service (16, 17), and the criteria for eligibility for support for autism remain inconsistent across cases as well as unclear (16).

The importance of understanding how Autistic people interact with the CJS is beginning to emerge. The National Health Service (NHS) in the United Kingdom (UK) has released a national strategy for Autistic children, young people, and adults and contains a section dedicated to the interactions of Autistic people and the CJS (18). The intention is to promote research about these interactions and improve the support for Autistic people within the CJS (18). Racialized Autistic peoples in Canada have unique interactions with the Canadian CJS (C-CJS), due to the historical context and legacy of colonialism and racism (19–22). Although CJS interactions with each identity have been studied, to varying extents, studies of the interactions of these intersecting identities are non-existent. Currently, there is an emerging acknowledgment of this lack of research, however, substantial, peer-reviewed articles on the topic continue to be lacking. In reviewing the literature, studies can be found on (i) Black and Indigenous peoples and the C-CJS, (ii) Autistic individuals and the C-CJS, and (iii) the intersection between Autistic and racialized identities.

The Canadian Charter of Rights and Freedoms established the freedoms and rights of every Canadian, regardless of race, gender, age, or background (23). However, the C-CJS has been reported to not uphold this unbiased testament (24), operating with the legacy of colonialism, and the context of racism (19, 20, 22). Black and Indigenous people have higher rates of interactions throughout the CJS such as homicide victimization and offending, police interactions, and incarceration rates (19). Rates of homicide victimization are much higher in Black and Indigenous populations, compared to the overall Canadian population (19). The Indigenous murder rate in Canada is almost seven times higher than the murder rate for non-Indigenous individuals (25). Indigenous adults account for 5% of the Canadian adult population yet represent 30% of the federally incarcerated inmates (26).

Similarly, people with neurodevelopmental dis/abilities, such as autism, are more likely to come into contact with the CJS when compared to their neurotypical counterparts (27). By the age of 21, approximately 20% of Autistic youth will have interacted with law enforcement officers (28). This can be in the form of offender, victim, suspect, or witness (27, 29), though it is important to note that Autistic people are more likely to be victims of violent crime instead of offenders (29). Characteristic behaviors of autism, such as being non-responsive or lack of eye contact, can increase the risks during interactions with the CJS as these behaviors can be perceived as intentional (28). Misinterpretation of these characteristics can result in the Autistic person being identified as dangerous, suspicious, or unreliable (28, 30).

The scarcity of research on racialized Autistic people is an important gap to address when we are discussing how Autistic and Black or Indigenous identities interact with the CJS. Autistic people are more likely than their neurotypical peers to have police interactions, and Black and Indigenous people are more likely to have negative outcomes of interactions with the CJS, in which they are overrepresented (19, 27). If the identities of racialized Autistic people continue to be separated when researching interactions with the CJS there can be no future planning, interventions, or policies that are culturally competent and effective for helping this unique community.

## Canada's National Autism Strategy

In Canada, most recent estimates have 1 in 50 children and youth in Canada diagnosed with autism, with more than 50% of those being diagnosed by the age of 6 and over 90% of children diagnosed by the age of 12 (31). It is recognized that these data may not be representative of Indigenous children with autism living in Canada (7, 32). In 2019, the Canadian Government committed to developing its very first NAS (33, 34). Autism strategies can be found in countries such as New Zealand, Malta, Australia, Spain, Scotland, Hungary, the USA, England, Wales, and Northern Ireland (35). In Canada, the NAS falls under the responsibility of the federal government (36). It is critical that Indigenous nations and the Canadian government develop a distinct approach for autism care, addressing the risks of jurisdictional disputes as well as creating an Indigenized approach. This is especially important when highlighting Article 19 of the Calls for Actions by the Commission of Canada's Truth and Reconciliation Commission (TRC) (37) as well as Articles 19, 21, and 23 of the United Nations Declaration on the Rights of Indigenous Peoples (38). For the elaboration of a NAS that is inclusive, it is imperative that a rights-based approach be used. This approach is recognized internationally; for instance, the United Nations Convention on the Rights of Persons with Disabilities and associated United Nations Committee on the Rights of Persons with Disabilities issued Concluding Observations on Canada's initial report (38) include specific recommendations to adopt cross-sectorial strategies to combat inequities and discrimination faced by persons with dis/abilities (39).

These highlight the importance of increasing our understanding of the experiences and needs of Autistic people who are Indigenous, especially in policy and practice. Unfortunately, research focused on intersectionality between race and autism is sparse, but critical to develop, as people with autism cannot separate their identities. The experience of a racialized Autistic person is unique and distinct in and of itself, especially when it involves the CJS.

## Our aim, objectives and intended uses

The goal of this Policy Practice Review is to provide a framework for the discovery of appropriate care strategies addressing the conceptualization of autism in Indigenous Peoples and to understand the interactions between racialized Autistic peoples and the Criminal Justice System. We scanned for publicly available autism supports and services with the purpose of highlighting issues and recommendations regarding the silos of support for Autistic people and families across Canada, with a focus on issues experienced by Indigenous children and youth on the spectrum, including jurisdictional disputes for care.

We used the Dis/ability Critical Race Theory (DisCrit) framework when presenting the support, issues, and recommendations highlighted in this scan, as this framework aids in understanding how the identities of racialized Autistic communities influence their interactions with Canadian systems. This worldview informed us to use identity-first language, such as Autistic person, rather than person-first language such as person with autism. Identity-first language has been indicated as the preferred language by most Autistic self-advocates (40). As one of the DisCrit tenets is that it privileges the voices of marginalized populations, traditionally not acknowledged within research, it was essential that we listened to the voices of Autistic self-advocates on their choice for preferred language (41). However, we acknowledge that Indigenous identity is complex and contextual. Meaning, the preference on language pertaining to the colonial definition of autism is context dependent and may differ among Indigenous communities.

DisCrit Theory informed the terminology choice in our discussions about Black and Indigenous peoples' interactions with the CJS. Within our research methodology for investigating the CJS the term BIPOC (Black, Indigenous, People of color) was utilized as a search term, as this term has become popular in some activism spaces, and greatly increased our likelihood of finding information on our population of focus (42, 43). However, BIPOC is not the preferred term of these communities, as it clusters communities and puts Black and Indigenous people at the center of any issues involving people of color (42, 43). This can result in distraction from the systemic issues, or from the accurate representation of how these communities interact with systems (42, 43). The term racialized allows us to focus on the systems and structures that create race and racialized people (those who deviate from the accepted norm, whiteness), and how the multiple systems and services affect these communities (44, 45).

Finally, this tenet informed our decision to not restrict our systematic literature reviews to peer reviewed articles. Racialized and Autistic voices have not had a dominant role in research, and their voices could be neglected in this search had we included this criterion. Therefore, to gain a broader understanding of the current situation for Indigenous and racialized Autistic peoples, it is important to listen to these voices in the alternative formats in which they have been able to speak, including magazine articles, news reports, interviews, and other forms of information sharing that are meaningful to these communities can allow a greater opportunity for these voices to be accurately conveyed, than if our systematic literature review was restricted to peer-reviewed articles.

Using this approach, we set out to answer two questions. First, we aimed to understand Indigenous people's perceptions and conceptualizations of Autism. Second, we aimed to understand the interactions between racialized Autistics and the CJS. Both questions had the purpose of informing policy and systems. To answer these questions, we facilitated two environmental scans, specified to each research question. Environmental Scan 1 focused on the perspectives and experiences of Indigenous Peoples in Canada with autism and dis/ability. Environmental Scan 2 focused on how Indigenous and Black communities uniquely interact with Canadian systems and supports.

## Methods

### Dis/ability critical race theory framework

To lessen the separation of intersectional identities that affect interactions with health, social systems and the justice system; the DisCrit framework is instructive in understanding this intersection. This framework can be used to understand why these identities cannot continue to be evaluated separately and frame how we can collect and assess data. DisCrit theories state that “racism and ableism are normalizing processes that are interconnected and collusive” [(41) p. 6]. Racism and ableism are grounded in the belief that deviations from a social norm, such as Black, Indigenous, or neurodivergent people (e.g., Autistic), are less valued (25). DisCrit emphasizes that these identities, and their correlated deviations from ideologies, must not be evaluated separately, as the systems themselves are interconnected (41). This highlights the importance of exploring the dearth of research on these interconnected identities, otherwise understood as the concept of intersectionality. Intersectionality is a theoretical framework encompassing unique identities such as race and ethnicity, gender, socio-economic status, and sexuality, and how they interact to affect a person's experiences in society (46). Historically, social endeavors such as slavery, segregation and employment justified these ideologies and current social structures for education, health, and justice may continue to perpetuate these ideologies (25). DisCrit Theory enables the examination of the intersection of identities for an Indigenous Autistic person, further, in the specified example of how racialized Autistic persons uniquely interact with the Canadian CJS (C-CJS). This information will enable the creation of policy and practices that improve interactions and outcomes of interactions with social systems, such as health, school, and the CJS, for Indigenous Autistic people.

### Environmental scans

Each environmental scan was composed of three phases: (1) we defined the context using a brief systematic literature review of scholarly articles for both the perceptions of autism in Indigenous communities, and the interactions of Indigenous and other racialized youth with autism with the justice system; (2) we scanned government websites for current programs and services targeting Autistic individuals and their families for both areas; and (3) we co-authored this review with two stakeholders. One was Grant Bruno, a PhD in Medical Sciences student at the University of Alberta. He is also a registered member of Samson Cree Nation, one of the four reserves that makes up the nehiyawak (Plains Cree) community of Maskwacis. He is also the father to an autistic son and currently chairs the Indigenous Relations Circle for the Autism Society of Alberta. The other, Carolyn Tinglin is a PhD candidate and emerging scholar in anti-Black ableism studies. Tinglin's work examines how race, dis/ability, and other identity-based social constructs intersect and impact racialized people. Both had relevant lived experience regarding the current policy environment. Here, stakeholders are defined as those with a vested interest in creating positive change for both the autism community and Indigenous Peoples, and contribute to the cause through experience, knowledge, and expertise.

A Strengths, Weaknesses, Opportunities, and Threats (SWOT) analysis was used to assess how political change, such as elections and varying legislation among provinces, impacted our policy scan (38). This has been previously used by Namugenyi et al. to identify and analyze the internal and external factors that have an impact on the viability of a project, product, place or person entities (47).

## Environmental scan of the perspectives and experiences of Indigenous peoples in Canada with autism and dis/ability

### Scholarly literature review

#### Procedure

A literature review was conducted to identify scholarly literature directly relevant to the beliefs and perceptions of autism and dis/ability within Indigenous communities in Canada. The search strategy included the key search terms “Indigenous,” “First Nations,” “autism,” “dis/ability,” and “Canada.” Indigenous Peoples search filters established by the University of Alberta and the University of Prince Edward Island were used to develop the search strategy for this review (48, 49). Systematic literature searches were performed in the following eight databases: Medline, Cumulative index to Nursing and Allied Health Literature (CINAHL), Web of Science, Scopus, PsychInfo, Arctic Health Publications Database, Circumpolar Health Bibliographic Database, iPortal, and Native Health Database (50). The titles and abstracts of all returned articles were screened thoroughly by the researcher for relevance to the research question. Included articles then underwent full-text review to ensure fulfillment of inclusion criteria (see Table 1). Themes related to the beliefs and perceptions of autism and dis/ability within Indigenous communities in Canada were extracted and gathered for further analysis.

**Table 1:** Inclusion and exclusion criteria for scholarly literature review on the beliefs and perceptions of autism within indigenous communities in Canada.

**Inclusion criteria**	**Exclusion criteria**
Literature on ideas, perceptions, values, beliefs, feelings, attitudes, practices, experiences and/or understandings of Autism and disability, through an Indigenous lens	Literature relating to Indigenous communities outside of Canada
Available in the English or French languages	Secondary research sources
	Duplications

#### Results

**Figure 1:**
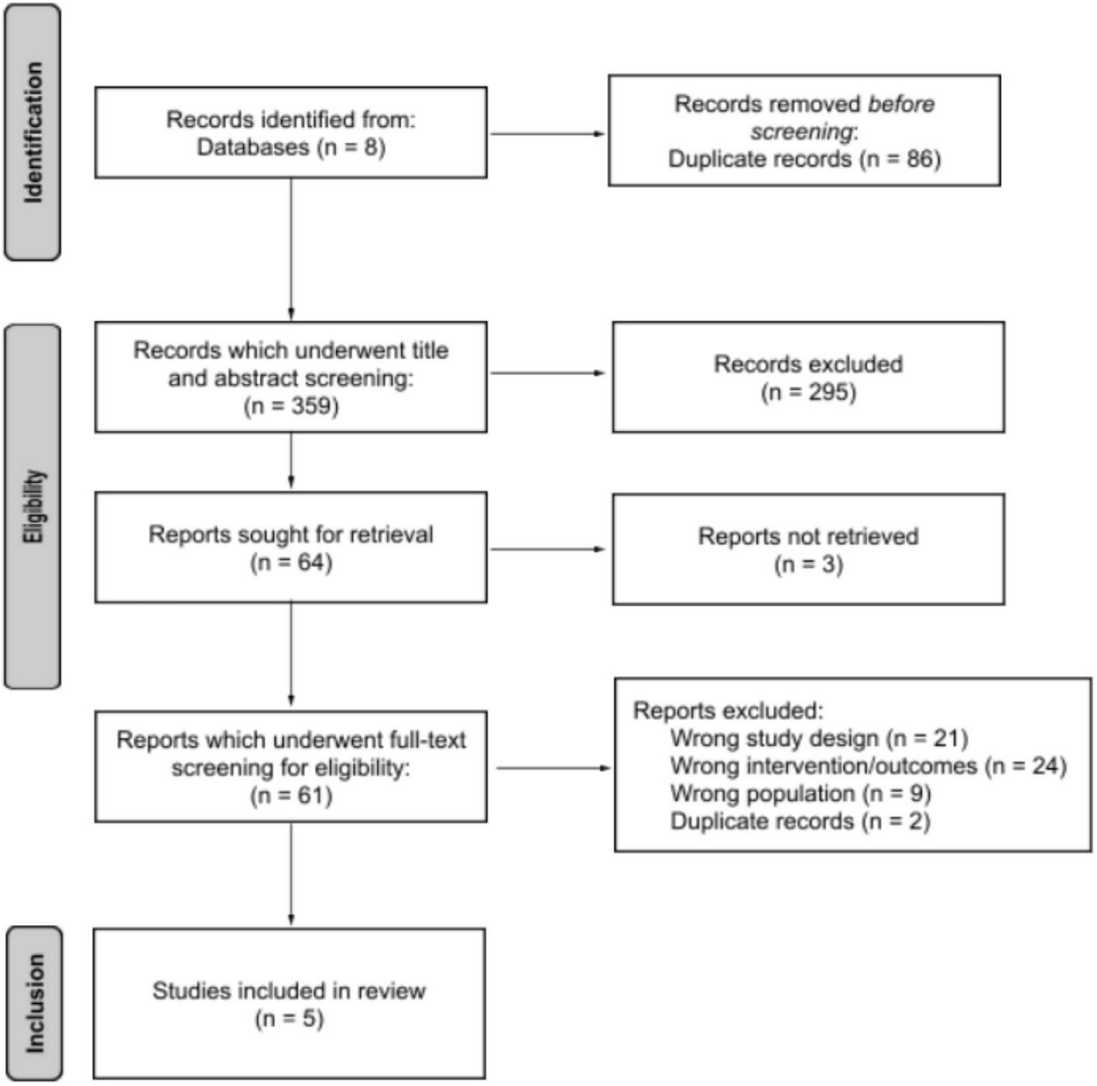
Systematic literature eligibility results for the perspectives and experiences of Indigenous peoples in Canada with autism and dis/ability. Adapted from: Page (51).

There was a total of 445 returned publications. The titles and abstracts of all returned publications were thoroughly screened, which led to the exclusion of 381 articles. Of these excluded articles, 86 were duplicates and 295 were on topics unrelated to the research question. The remaining 64 publications then underwent full-text review, after which there remained a total of five articles included for review (see Figure 1). The five articles in this review exhibited the following themes:

##### Theme 1: Inclusion and acceptance

The theme of inclusion and acceptance demonstrates the warmth with which Indigenous Peoples embrace autism and dis/ability. In some Indigenous cultures, autism is not conceptualized as a form of deficit or shortcoming (52). Rather, it is acknowledged and accepted by community members, with the intent to support the individual to their strongest capacity (52–54). This contrasts with the medicalized, deficit-model employed within Western societies, which labels and treats the individual's condition as a medical issue requiring treatment intervention. To our knowledge, Indigenous Peoples often do not label or categorize community members based on their abilities (52). Labeling may evoke a sense of division among community members, which can cause strain in community relationships. Alternatively, many Indigenous communities treat Autistic or dis/abled people as any other valued member of the community, and they receive the support of their family and community members in their daily lives (52). In sum, Indigenous communities in Canada foster a climate of inclusion and acceptance of the diverse contributions of all community members, despite their unique abilities.

##### Theme 2: Supportive network of family and community members

The theme of a well-supported network of family and community is articulated across all five of the articles included in this review. Indigenous ways of living often emphasize the interconnectedness of family and community, promoting a sense of inclusion and acceptance of people of all abilities (53, 55). When defining dis/ability, it was demonstrated that some Indigenous communities place emphasis on the community context (55). Dis/ability can be considered to impact not merely the individual themselves, but the family and community with which they interact (55). Once a dis/ability is recognized, it is embraced by community members and can become the shared responsibility of the individual's family and community to help provide a vital source of support (52–54). This close-knit network of family and community can provide a powerful sense of inclusion and empowerment to the Autistic or dis/abled person.

Furthermore, within some Indigenous communities, dis/ability can be viewed as a result of disharmony or imbalances in aspects of the mind, body, and spirit (55, 56). Dis/ability can also be understood to manifest due to imbalances within various environmental contexts, such as family, school, and the greater community (55). This reflects the holistic approach that is habitually employed within Indigenous communities to assess health and wellbeing. Thus, strengthening one's connections with family, school, and community can help to restore a sense of harmony and balance in people with dis/abilities, optimizing their overall health and wellbeing (55).

##### Theme 3: Engaging in Indigenous cultural identity

The theme of engaging in Indigenous cultural identity to optimize wellbeing conveys the need for culturally safe supports and services for Indigenous Peoples with autism and dis/ability. To our knowledge, the implementation of Indigenous ways of knowing, being, and doing within services can help to support the desire of Indigenous Peoples with dis/abilities to partake in traditional culture in a meaningful way (56). Practicing one's Indigenous cultural identity, through the teaching and learning of cultural traditions, can serve as a protective factor in conserving the wellbeing of Indigenous Peoples, both individually and collectively (56).

In Lindblom's case study involving five Autistic First Nations children in British Columbia, the meaning of music to the children was examined (52). The study findings indicated that there was a lack of cultural sensitivity in the music interventions allocated for Autistic First Nations children in British Columbia (52). Lindbolm's following article highlighting two of these cases explored the intersections of dis/ability, gender, ethnicity, and culturally safe traditional music interventions in the improvement of communication and social inclusion among Autistic First Nations children (53). Lindbolm's findings suggested that meaningful engagement of Autistic First Nations children in traditional practices, such as Indigenous music interventions, can help to ease personal challenges and enhance communication and social development (53). Hence, the implementation of supports and services that are culturally safe may help to strengthen Indigenous cultural identity and support the development of Indigenous children with autism.

### Gray literature scan

A gray literature scan was performed to assess all of the existing policies and programs being delivered within Northern Indigenous communities in Canada, specifically in the territories of Nunavut, Yukon, and the Northwest Territories (NWT). Following this gray literature search, a S.W.O.T. analysis was performed to critically comment on the programs and services available in the North, in terms of their strengths, gaps, and areas for improvement (see Table 2).

**Table 2:** Summary of S.W.O.T analysis for gray literature search on the perspectives and experiences of indigenous peoples in Canada with autism and dis/ability.

**S.W.O.T components**	**Indigenous perspectives on autism**
Strengths	Programs and services incorporate autism within the broader lens of “disability.” This use of terminology can minimize the likelihood of labeling, which can be detrimental to Indigenous communities.
Weaknesses	There is a lack of programming specified toward Autistic people or Indigenous Autistic people. There are only two disability-focused programs in Yukon, the Home Care Program, and the Home Repair Program, which include First Nations residents in their eligibility, but do not have services specified for their needs. Further, the language used in program information reflects person-first language, which portrays disability as a deficit and may misalign with Indigenous cultural views.
Opportunities	There is an evident need for culturally safe programs, developed in collaboration between the Canadian government and Indigenous Peoples within the context of the NAS. Additionally, changing to identity-first language can reduce the stigma of disability and better reflect Indigenous cultural views for those seeking support.
Threats	There is a dearth of research focused on autism in Indigenous communities in Canada. Existing studies have found significant underrepresentation of Indigenous Peoples diagnosed with autism in reports of autism prevalence. This lack of research could result in a lowering of prioritization for the Canadian government, resulting in a continued lack of culturally safe support for Indigenous Autistic people. Moreover, limited internet access in the North may reduce online availability of pertinent information for the advancement of policies and programs.

### Recommendations for decision-makers in developing autism supports for Indigenous peoples

#### Actionable recommendations

Although Indigenous Autistic people in Canada experience some of the same challenges as non-Indigenous Autistic individuals, it is important to recognize and understand the complex layers and intersections colonialism has created. The following recommendations are meant to be approached with a distinction-based focus, meaning that there is a need to recognize the diversity and uniqueness of each group including differences for First Nations, Métis, and Inuit, as well as geographic differences such as urban, rural, and remote as well as differences in culture and language. It should also be noted that these recommendations are not comprehensive and as the NAS is implemented new priorities may arise. One more thing to consider is some Indigenous communities' challenges need to be addressed upstream. For example, some communities may have challenges around poverty, chronic housing shortages, boil water advisories, etc. that may need to be addressed at the same time as addressing the challenges around autism. Using an approach that allows the community to lead the project in full partnership is now considered best practice. The following are actionable recommendations to address the aforementioned issues and themes:

1. Develop an Indigenous autism engagement framework that recognizes and honors the diversity of Indigenous peoples across Canada. Build relationships with key Indigenous individuals and groups using a distinction-based approach by creating national autism advisory groups for First Nations, Métis, and Inuit, respectively.
2. Assess the barriers to healthcare including diagnostic assessments, services provision, socio economic considerations, and overall mistrust of the health care system. Areas to focus on include racism, ableism, poverty, and current and historical injustices. An actionable step would be to, in partnership with Indigenous groups, develop a report that focuses on the barriers and solutions to accessing healthcare.
3. Partner with Indigenous communities and organizations to collect data and evidence to understand autism prevalence and the lived experience of autism in Indigenous communities. Listen to Indigenous Autistic people in the ways that they wish to be heard, to ensure the collection of data and evidence that is both meaningful and accurate to Indigenous communities. Provide support for Indigenous-led research in partnership with communities and organizations to create empirical evidence that reflects the wants and needs of Indigenous Peoples in Canada.
4. Explore what Indigenous led and culturally safe services, assessments, and interventions would look like. In full partnership with Elders, traditional knowledge keepers, community members, Indigenous autistic individuals, and Indigenous service providers, create and pilot culturally-informed autism services.
5. Provide equitable funding across Canada for Indigenous communities and organizations to provide culturally safe autism services, appropriate autism assessments, autism awareness and education. Research and develop a report that addresses jurisdictional disputes between federal, provincial, municipal, and band councils that are specific to autism in Indigenous communities and explore opportunities to address these challenges.

## Environmental scan of how Indigenous and Black communities uniquely interact with Canadian systems and supports

### Scholarly literature review

#### Procedure

Four Databases were searched: ProQuest, Web of Science, PubMed, and EBSCOhost. Truncations were implemented to retrieve articles that contained multiple applications of the word stem, in order to broaden the search and generate as many relevant articles as possible. The search terms pertained to three domains to explore the research pertaining to racialized Autistic individuals and the CJS (see Table 3). The domains included autism, Black and Indigenous peoples, and the Justice system. Each domain contained several possible search terms that could attempt to address the domain, connected by the Boolean operator “OR.” Each of the three domains was connected with the Boolean operator “AND.” The final search terms were applied to each database: (autis^*^) AND (crim^*^ OR police OR arrest OR charge OR justice OR court OR law) AND (black OR indigenous OR aboriginal OR first nations OR métis OR inuit OR rac^*^ OR minority OR intersect^*^).

**Table 3:** Search terms for racialized autistic individuals and the CJS.

**Domain:**	**Autism**	**Justice** **system**	**Black and** **Indigenous peoples**
Search terms:	Autis^*^	•Crim^*^ •Police •Arrest •Charge •Justice •Court •Law	•Black •Indigenous •Aboriginal •First Nations •Métis •Inuit •Rac^*^ •Minority •Intersect^*^

* Refers to the truncation of a search term, which is the shortening of a search term in a literature search to attract words with the same root word, but different endings.

#### Results

**Figure 2:**
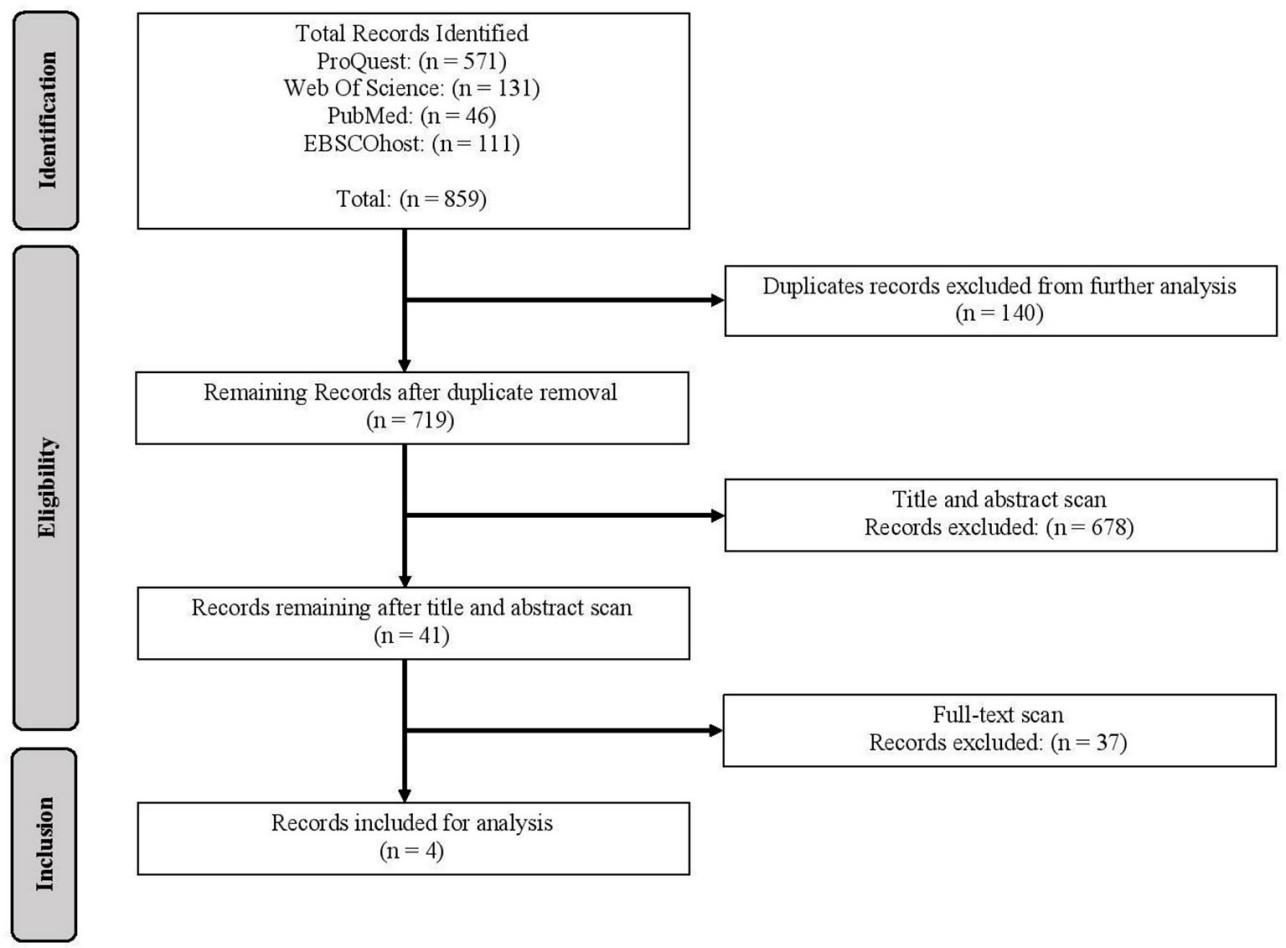
Systematic literature eligibility results for how Indigenous and Black communities uniquely interact with Canadian systems and supports.

In total 859 publication records were collected from the four databases. The publications were then scanned based on inclusion and exclusion criteria (depicted in Figure 2). The inclusion and exclusion criteria (depicted in Table 4) focused on ensuring that relevant, recent publications addressing the intersectionality of identities for racialized Autistic individuals interacting with the CJS were included for final assessment. The publications were first scanned for duplicates; 140 publications were excluded from further analysis. A title and abstract search were then conducted using the inclusion and exclusion criteria, after which 678 articles were excluded. Lastly, based on this same criterion, a full text scan was conducted; 37 articles were excluded from the qualitative analysis. In conclusion, after applying the inclusion and exclusion criteria 855 publications were excluded, and four publications were included in the final qualitative analysis of the systematic literature review.

**Table 4:** Inclusion and exclusion criteria for scholarly literature review on racialized autistic individuals and the CJS.

**Inclusion**	**Exclusion**
Researcher has access to the full article	Researcher does not have access to the full article
Published in the English or French language	Published in languages other than English and French
Unique publication	Duplicate of another publication
Article's subject centers on how each of the three domains of the research question intersect and affect each other. Articles will do this through discussing at least one facet of each of the three domains: (1) BIPOC (2) autism (3) criminal justice system In order to assure that the main research question is able to be answered: What are the outcomes of Autistic Black and Indigenous youth's interactions with the Justice System	Article's subject does not center on the intersection of each domain, that needs to be considered while aiming to answer the question of how the intersecting identities affect interactions with the criminal justice system.
Article focuses on Autistic people	Article does not make clear whether Autism qualifies in their definition of a developmental disability; the article discusses dis/abilities or other neurodevelopmental disorders that are not autism, e.g., FASD instead of ASD.
The subject of the article is focused on youth; the publication depicts multiple life stages which includes youth	The subject of the article excludes youth, focusing on adults or elderly people.
The subject of the article is non-fictional	The subject of the article is fictional

All articles included were based in the United States of America (USA) and focused on Black Autistic people's interactions with the CJS. Articles geographically based outside of the USA or focusing on Indigenous Peoples were not found during this literature search or were not applicable to the inclusion and exclusion criteria. The four articles examined had three main themes:

##### Theme 1: Increased risk of interactions for racialized Autistics with the CJS

The theme of increased risk of interactions for racialized Autistics with the CJS explores how intersecting identities can lead to and prolong interaction with the CJS. For example, having racialized identity can prompt interaction with police, such as the situation explored by Vargas, where the characteristics and presentation of autism (such as aversion to touch) can escalate an interaction with police and lead to negative outcomes (57). This trend of one identity initiating the interaction, and one identity increasing the risk and negative outcomes continues throughout this literature search. Carley, as well as Solomon and Lawlor explored how the characteristic behaviors (such as elopement) and fixations of autism can lead to police interactions, during which being a racialized Autistic person increases negative outcomes (58, 59). Meaning, further prejudice toward the racialized Autistic person, misunderstanding of characteristic behaviors, and continued negative outcomes with a lack of support for the individual (58, 59). Davenport et al. documented a program to train Autistic Black adolescents on how to interact with police (60). Davenport et al. recognized the increased risk of interactions for both identities (without research specified to the intersection of identities), and negative outcomes from individuals within the CJS's notions and conceptions of either identity (60).

##### Theme 2: Lack of culturally competent supports and interventions

The theme of lacking culturally competent support and interventions focuses on how systems fail to adequately support racialized Autistic youth at multiple stages of interactions with the CJS. Meaning, that systems lack cultural competence in understanding how racialized Autistic persons uniquely interact with the CJS in terms of prevention of crime, arrest, charging, court appearance, and incarceration. This could be due to a lack of understanding on how characteristic behaviors of autism (such as fixation, and elopement) can lead to interactions with the CJS, and/or the lack implementation of supports which could act as preventative measures such as demonstrated by Carley as well as Solomon and Lawlor (58, 59). Carley then further explored how Darius McCollum was treated differently in the courts due to his intersecting identities of being Black and Autistic, which led to harsher sentencing and misunderstanding of the locus of control (58). As the judge perceived Darius McCollum's actions to be due to solely his own volition and control, rather than a continued lack of support, and systemic misunderstanding of Autism, this led to continuing the sentencing of jail time, and the continued lack of support for coping with this fixation which led to criminal behavior (58). At the initial interaction police are inept to interact with Autistic people and the manifestation of autism characteristics and behaviors, leading to escalating situations. Especially when considering the prejudices that police enter the situation with if the Autistic person is Black or Indigenous, as explored by Vargas (57). The study and program created by Davenport et al. was a response to this lack of culturally competent support and interventions (60). Their goal was to not only recognize the unique interactions Black Autistic persons have with police, but also to train these adolescents on how to navigate this interaction (60).

##### Theme 3: Uninformed and biased systems

The final theme of uninformed and biased systems describes how the CJS fails racialized Autistic youth at every step, due to a lack of knowledge, prejudice, misinformation, racism, and ableism. At initial interaction police are ill equipped to interact with Autistic people and their manifestation of characteristics, leading to escalating situations. This is especially true when considering the prejudices, the police begin the situation with if the Autistic person is Black, as the results of this literature review focused on. This was explored by Vargas as the scenario was initiated when the police were called because a Black man appeared suspicious while sitting down waiting outside the library (57). The interaction is believed to have escalated further due to both the characteristics of autism, and the Black identity of the man (57). Furthermore, Carley explores how the combined identities of being Black and Autistic led to harsher, inappropriate sentencing, and a continued lack of support, due to prejudice and racism (58). For example, the judge demonstrated a lack of understanding and prejudice of autism as the judge was quoted as saying that Asperger's syndrome (a previous term used for diagnosis of autism) was a “dangerous, mental disorder” (58). This prejudice is not restricted to the CJS, as explored by Solomon and Lawlor where, when Black mothers would reach out to healthcare teams for support with their children's elopement, they were often dismissed for their concerns (59). This is a trend not seen in their white counterparts (59). This dismissal provides no support for the behavior, which in turn can lead to CJS interaction and involvement, potentially putting children at risk of further negative outcomes (59). Finally, these inefficient and biased systems can lead to communities creating support systems in response, as was explored in the creation and implementation of the program explored by Davenport et al. (60). Davenport et al. recognized that Black Autistic adolescents are too much at risk to ignore how these intersecting identities disproportionately result in the risk of negative outcomes (60).

### Gray literature scan

Each Province and Territory's government website, as well as the federal government websites, were scanned for resources that Autistic people and their support systems could access when interacting with, or having interacted with, the C-CJS. Following this gray literature search, a S.W.O.T analysis was performed to critically comment on the programs and services available in Canada, in terms of their strengths, weaknesses, opportunities, and threats (see Table 5). Two provinces and two territories, Ontario, Alberta, Yukon, and Northwest Territories indicated some support available for people who are involved with the C-CJS in some capacity. In analyzing the descriptions of available support on ministry websites, the depth of information was restricted to short descriptive texts, ranging from indicating access to support through this program, assurance of continued human rights, or indicating availability of community programs. For example, Ontario indicates that Children's and Young Person's rights remain recognized, even when the youth is in the justice system. Alberta promises specialized support from experts to help if someone with developmental dis/abilities has additional needs because of mental illness, behavioral issues, addictions and/or involvement with the law. The Yukon has defined two separate programs. The first is for offenders and is based on changing behavior by targeting antisocial thoughts and cognitive skills deficits. The second collaborates with Yukon First Nations to incorporate Indigenous cultural heritage into the correction processes. The NWT has created programs designed to address the underlying issues that may contribute to reoffending.

**Table 5:** Summary of S.W.O.T analysis for gray literature search on how indigenous and black communities uniquely interact with Canadian systems and supports.

**S.W.O.T** **components**	**Racialized Autistic's interactions with the** **CJS**
Strengths	The need for specialized support for Autistic people, relative to the CJS, has begun to emerge. Meaning, some provinces and territories have begun to implement programs for CJS support for Autistic people, or statements regarding continued human rights.
Weaknesses	Not every province and territory has acknowledged this need, meaning, inconsistency in support available across Canada. Acknowledgment of intersectionality necessitating specified care was not found.
Opportunities	The NAS provides an opportunity for incorporation of specified supports for intersectional identities that are consistent across Canada. Further, the Specialized Court: Wellness Court in the Northwest Territories provides an example for future development.
Threats	As research on racialized Autistics, such Black and Indigenous people, is greatly lacking, this could result in programs specified to this population not being prioritized by the Canadian government when developing a NAS. Meaning, the Canadian government might choose to focus on populations and issues that have a greater wealth of information on needs, resulting in programs' continued failure in supporting racialized Autistics in interacting with the C-CJS.

### Recommendations for decision-makers in supporting racialized autistic people with the criminal justice system

#### Actionable recommendations

The following are actionable recommendations to address the issues and themes discussed relative to racialized Autistic people and the C-CJS:

1. Commit resources including funding, technology, and human resources to develop research examining the ways in which Canadian policing policies and practices impact racialized youth with autism. Creating this research opportunity is imperative as there are negligible current research studies that investigate how justice system policies and practices impact racialized and Autistic people.
2. Ensure racialized Autistic people are central to research focused on race, dis/ability, and policing. Furthermore, ensure equitable opportunities for racialized people with autism to meaningfully engage in this area of research, recognizing the importance of community-based research for and by community members.
3. Review and/or develop policing policies which address anti-Black racism, as well as racialized ableism, while supporting equitable care of racialized Autistic people in carceral settings. In other words, create policies and practices grounded in anti-racism and anti-ableism, ethics of care, and culturally competent protocols which guide encounters with the justice system.
4. Establish a mandatory training program for law enforcement, incorporating the experiences and knowledges of racialized Autistic people, grounded in anti-racist, anti-ableist approaches to policing.
5. Be proactive in identifying opportunities for justice system stakeholder engagement being mindful of the historical, political, and social contexts and implications of policing minoritized Canadians.
6. Create accessible services for racialized Autistic people who are incarcerated, based on individual and community needs. This might be accomplished through community round tables, stakeholder forums, and justice system policy reviews.
7. Implement an anonymous *police encounters reporting system/hotline* to collect important data about law enforcement encounters with racialized Autistic peoples across Canada.
8. Provide additional resources and funding to agencies which serve racialized youth with autism, to research and address the psychosocial, physical, and emotional wellbeing of intersectionally-positioned youth who are incarcerated.

## Discussion

In this paper, we synthesize findings from both the perspectives and experiences of Indigenous Peoples in Canada with Autism and Dis/ability and how Indigenous and Black Communities uniquely interact with Canadian systems and supports in order to clearly present commonalities and develop policy and practice recommendations. To understand the data and recommendations presented in this article, it is essential to maintain understanding of the historical, and current context within which Indigenous Peoples in Canada live. Indigenous Peoples in Canada live within the historical context, and continual legacy, of colonialism and systemic oppression. This has taken forms such as the Indigenous Residential School system, the sixties scoop, the implementation of the Indian hospitals, and continual neglect of basic needs, such as clean water on reservations (10–12, 61). This historical, and current, context and events have had detrimental effects on Indigenous people's perceptions and trust of Canadian systems and supports and thus lead to negative health outcomes (5).

This context further extends into understanding how Indigenous Peoples, specifically Indigenous Autistic people, uniquely interact with Canadian systems and supports. The two main systems explored in this article, diagnostic services and the CJS, both have roots in colonialism and institutional racism which continue to affect Indigenous communities, and other racialized groups such as Black communities (13, 19). This can be seen in terms of a misalignment of conceptualization of autism, underdiagnosis, and supports and systems that fail to acknowledge and implement Indigenous ways of knowing (14). Furthermore, this can also be seen in the overrepresentation of Indigenous Peoples in the CJS (19, 26). This historical, and current context allows for better understanding on why these systems are currently ineffective, and inadequate at supporting Indigenous communities within Canada, specifically related to the topic of autism.

To ensure that Indigenous Peoples with autism have access to meaningful supports and services across the lifespan, it is crucial to implement culturally safe health and social services within Indigenous communities. Indigenous Peoples identify with distinct cultural definitions of wellness and experience unique interactions with the criminal justice system. Nonetheless, Indigenous Peoples are currently required to adopt Western perspectives on disability to receive adequate access to services and funding through the diagnostic process, which demonstrates the continued colonialism, institutional racism, systemic barriers with which the health and criminal justice systems are embedded. Government programs and policies often adopt a Western, deficit-model approach to assessment tools and services, which misaligns with Indigenous cultural values and practices. Appropriate care and social service pathways for Indigenous Peoples with autism can be developed through strategies that acknowledge the conceptualization of autism within Indigenous communities and the unique challenges that Indigenous Peoples face across the lifespan.

The findings from the included systematic literature reviews reveal the growing gaps in research regarding autism in Indigenous communities in Canada and how racialized Autistic Peoples interact with the CJS. With a total of five articles included for the former area of study and four articles identified for the latter, several common themes surfaced across the literature with regards to the perceptions of autism in relation to Indigenous Peoples.

In Indigenous communities in Canada, autism is acknowledged with acceptance and is received with inclusion and empowerment by community members. Indigenous cultures are distinctly welcoming of diversity and embrace the unique abilities and contributions of all community members. When an individual conveys specific needs, community members can take up the instinctive responsibility to help them perform in their roles to the best of their capacities (52–54). This lies in direct contrast to the perceptions of autism demonstrated by individuals in the CJS. The Judge trying Darius McCollum stated that Asperger's syndrome (a previous term used for diagnosis of Autism) was a “dangerous, mental disorder” (58). Solomon and Lawlor continued this discussion of how perceptions can impede gaining adequate support and fair treatment relative to the CJS (59). When Black mothers would seek help in addressing elopement (a behavior that can present in Autistic people), they found their concerns were often dismissed and support in altering this behavior which could lead to CJS involvement was absent (59).

To our knowledge, dis/ability in some Indigenous communities is not an isolated experience. Rather, it has extensive impacts on the family and community with which the individual interacts (55). Indigenous Peoples hold a strong interconnectedness with community, as it serves as a critical source of support for Indigenous Peoples, especially those with autism and dis/ability (52–54). Thus, a disharmony in one's connections with family and the greater community may substantially influence Indigenous experiences with autism and dis/ability, especially when individuals try to seek support from systems outside of the local community.

When reviewing the system's cultural competency, relative to the CJS, there is a need for improvement particularly around a lack of understanding the characteristics of autism, and the potential to lead to interactions with the CJS (58, 59). Or, the lack of support which could prevent interaction with the CJS (58, 59). This is essential to recognize as being a racialized Autistic person can have risks when interacting with the CJS (57). This has led to communities being the ones to develop supports and programs in order to not only recognize that this is an issue, but also take steps toward creating solutions and supports (60).

Indigenous Peoples with autism and dis/ability often desire to engage in Indigenous cultural traditions and practices (56). However, there is a lack of culturally safe interventions and services available for Indigenous Peoples with autism (7, 52). This can lead Indigenous Peoples to feel disconnected from their cultural identity, which can pose detrimental impacts on the mental health and wellbeing of Indigenous communities (62). According to Lindblom, it was determined that the interactions of Autistic First Nations children with traditional practices, such as Indigenous music interventions, can help to ease personal difficulties and improve communication and social interactions (53). Hence, the incorporation of interventions and services that are culturally safe may help to strengthen Indigenous cultural identity and, as a result, enhance the development of Autistic Indigenous children.

Within our results, two key issues emerge. First, one of the main weaknesses found in the environmental scans was a lack of acknowledging how intersectional identities inform the need for specified programs and supports. Meaning, that there was scant mention of how Indigenous ways of knowing are incorporated into current supports and programs, nor how Indigenous conceptualizations of autism are taken into consideration. Furthermore, the implications of how the intersectional identities of racialized Autistics would affect interactions with the CJS, and resulting necessitated programs, were not found. Second, the threats portion of the S.W.O.T analysis provided further insight into the challenges that could arise while aiming to reduce this deficiency. Both the field of Indigenous perspectives on autism, and racialized Autistic interactions with the CJS are undeveloped. This could create a barrier when the Canadian government is deciding on what new programs to create, and how to create them. Meaning, they might be more likely to gather political support and gain funding if they choose to implement programs with a wealth of evidence on the likelihood of success, and dire need of implementation. Thus, this lack of research not only is an issue in and of itself, but it further creates a barrier when trying to develop and implement programs related to these fields.

To create programs that can appropriately support Indigenous autistic people, and address relevant issues the actionable recommendations are as follows:

1. The current barriers and systems need to be assessed with a historical context in mind. These areas include racism, ableism, poverty, current and historical injustices, as well as the current policies and practices related to policing. This is essential to future research and planning of policies and practices as without a context on the current situation, and what has led to its culmination, these new implementations could continue to be ineffective at addressing the needs and issues of Indigenous Autistic and other racialized communities.
2. It is critical that commitment is made to the development of strength-based Indigenous-led research partnerships and support provided for the synthesis of research that reflects the health and social needs of Indigenous Peoples living in Canada. Within research partnerships, it is imperative that opportunities for meaningful engagement are present and accessible to Indigenous communities throughout the research process. Ensuring that the entire process is Indigenous-led can allow for the performance of research that is both meaningful and applicable to Indigenous communities.
3. New culturally informed, and culturally competent policies and practices must be curated. These new policies must be grounded in anti-racism, anti-ableism, and ethics of care to adequately serve the communities they are being curated for. To do so, these interventions must be created and developed in partnership, in meaningful and sustainable ways.
4. Equitable funding and resources are required to adequately invest in the development of culturally safe and competent services, assessment tools, and programs for Autistic Indigenous Peoples. These services and programs will aim to address the psychosocial, physical, and emotional wellbeing and needs of intersectionality-positioned populations with Autism across the lifespan.

### Limitations

A critical gap exists in the literature with regards to Autism in Indigenous communities in Canada and how Autistic Indigenous Peoples interact with the CJS. The lack of Indigenous perspective of autism in Canada may derive from the over-emphasis of research on fetal alcohol spectrum disorder within Indigenous populations (63). It can be argued that the focus of FASD research further reinforces negative stereotypes of Indigenous people and is a form of victim blaming. In the absence of a comprehensive understanding of the various factors that shape Indigenous experiences with Autism and their interactions in seeking health and social services, the needs of Autistic Indigenous Peoples may emerge to be misrepresented in developing policies and programs. This emphasizes the need for further research in this area of study, to inform the development of culturally safe and competent services for Autistic Indigenous Peoples across the lifespan.

Another critical gap in data, pertaining to how racialized peoples interact with the criminal justice system, outside of the USA, affected our ability to create a holistic picture of how racialized Autistic people interact with the C-CJS. Although recommendations were formulated based on the literature reviewer and environmental scan, this limitation in the applicability of data is important to note.

We also recognize the limitation of using environmental scans of publicly accessible government websites to search for current programs and supports. This could have restricted the programs with which we were able to discover, as some programs might be in development, yet have not been listed publicly at the time of the search. Furthermore, we did not have access to in-depth descriptions of programs and were restricted to what was available to the public.

Additionally, it is of consideration that lack of critical infrastructure such as internet access may be limited in some of the Northern provinces and territories in Canada. This would have limited the information that we were able to retrieve, as other pertinent information relating to the current programs and services may have been publicized on alternative platforms beyond the online network. Some government websites were devoid of information on their last update. This could have also impacted the recency and relevance of the programs found, due to this missing information.

### Conclusion and future directions

There is still much work to be done to modify Canadian supports and systems to adequately care for Indigenous Autistic peoples. This Policy and Practice Review identified pertinent issues relative to these systems and their ability to provide culturally safe care, and prevent harm, specifically diving into the example of racialized Autistic peoples and the CJS. However, the fields of research related to autism within Indigenous communities in Canada, and how Autistic Indigenous individuals interact with Canadian systems are still in their infancy. Research should be based on continuous and meaningful engagement with Indigenous communities to assure that all changes made, and innovations proposed, are based in culturally safe and competent practices. Mutual relationship and collaboration with Indigenous Elders, traditional knowledge keepers, community members, Indigenous Autistic individuals, Indigenous service providers, and other relevant stakeholders is pivotal throughout the entirety of the research process, from the development of methodologies to the analyses of results. Partnerships with Indigenous communities in the collection of data and evidence that affects Indigenous Peoples should be Indigenous-led, to ensure that data collection and research is meaningful to Indigenous communities. Further, within this engagement there should be capacity building and training for Indigenous researchers, services providers, and policy makers, to assure continued culturally safe and competent practices extending beyond this initiative. This is essential, as with the described historical and current context within which Indigenous Peoples live, self-determined and Indigenous-led research and policy development through Canada's upcoming NAS can act as a method to overcome barriers due to historical neglect and abuse. Should the above recommendations be implemented, there could be a significant impact on how Indigenous Peoples in Canada define autism, what success looks like for supports, and mechanisms to focus resources and innovation.

There remains extensive work to be done in the development of adequate and meaningful supports that serve the needs of Autistic Indigenous Peoples and racialized communities in Canada. However, there are clear opportunities for developments in research and policy through the development and implementation of a NAS, and actionable steps that will allow for the achievement of equitable service access and culturally safe and competent care for such communities.

## Author contributions

CA and MC wrote the initial draft equally as shared first authors. GB and CT contributed academic and lived experience insight. JL and SC conceived of the presented idea and supervised the work. All authors discussed contributed and approved the final manuscript.

## Conflict of interest

The authors declare that the research was conducted in the absence of any commercial or financial relationships that could be construed as a potential conflict of interest.

## Publisher's note

All claims expressed in this article are solely those of the authors and do not necessarily represent those of their affiliated organizations, or those of the publisher, the editors and the reviewers. Any product that may be evaluated in this article, or claim that may be made by its manufacturer, is not guaranteed or endorsed by the publisher.

## References

[1] Indigenous Northern Affairs Canada. Indigenous Peoples and Communities. Government of Canada (2009). Available online at: https://www.rcaanc-cirnac.gc.ca/eng/1100100013785/1529102490303

[2] Minister of Justice. Indian Act. (2019). Available online at: https://laws-lois.justice.gc.ca/eng/acts/i-5/fulltext.html

[3] Indigenous Services Canada. About Indian Status. Government of Canada (2008). Available online at: https://www.sac-isc.gc.ca/eng/1100100032463/1572459644986

[4] Health Canada. Canada's Health Care System. Government of Canada (2011). Available online at: https://www.canada.ca/en/health-canada/services/health-care-system/reports-publications/health-care-system/canada.html

[5] Browne AJ Varcoe C Lavoie J Smye V Wong ST Krause M. Enhancing health care equity with Indigenous populations: evidence-based strategies from an ethnographic study. BMC Health Serv Res. (2016) 16:544. 10.1186/s12913-016-1707-9 27716261 PMC5050637

[6] Government of Canada. First Nations. (2008). Available online at: https://www.rcaanc-cirnac.gc.ca/eng/1100100013791/1535470872302

[7] Lindblom A. Under-detection of autism among First Nations children in British Columbia, Canada. Disabil Soc. (2014) 29:1248–59. 10.1080/09687599.2014.923750

[8] Ouellette-Kuntz H Coo H Yu CT Chudley AE Noonan A Breitenbach M. Prevalence of pervasive developmental disorders in two Canadian provinces. J Policy Pract Intellect Disabil. (2006) 3:164–72. 10.1111/j.1741-1130.2006.00076.x17088274

[9] George J MacLeod M Graham K Plain S Bernards S Wells S. Use of traditional healing practices in two Ontario first nations. J Commun Health. (2018) 43:227–37. 10.1007/s10900-017-0409-5 28861672

[10] Eric Hanson Daniel P. Games, Alexa Manuel. The Residential School System. Indigenous Foundations (2020). Available online at: https://indigenousfoundations.arts.ubc.ca/residential-school-system-2020/

[11] Eric Hanson. Sixties Scoop. Indigenous Foundations. Available online at: https://indigenousfoundations.arts.ubc.ca/sixties_scoop/

[12] McGregor D. Traditional knowledge and water governance: the ethic of responsibility. AlterNative Int J Indig Peoples. (2014) 10:493–507.

[13] Hill DM. Traditional medicine and restoration of wellness strategies. Int J Indig Health. (2009) 5:26–42. 10.3138/ijih.v5i1.28976

[14] Redvers N Blondin B. Traditional indigenous medicine in North America: a scoping review. PLoS ONE. (2020) 15:e0237531. 10.1371/journal.pone.0237531 32790714 PMC7425891

[15] Berkes F. Indigenous ways of knowing and the study of environmental change. J R Soc N Z. (2009) 39:151–6. 10.1080/03014220909510568

[16] Blackstock C. Jordan's principle: Canada's broken promise to First Nations children? Paediatr Child Health. (2012) 17:368–70. 10.1093/pch/17.7.368 23904779 PMC3448536

[17] Sinha V Wong S. Ensuring First Nations children's access to equitable services through Jordan's principle: the time to act is now. Paediatr Child Health. (2015) 20:62–4. 10.1093/pch/20.2.62 25838774 PMC4373574

[18] Government of United Kingdom. The National Strategy for Autistic Children, Young People and Adults: 2021 to 2026. (2021). p. 43. Available online at: https://assets.publishing.service.gov.uk/government/uploads/system/uploads/attachment_data/file/1004528/the-national-strategy-for-autistic-children-young-people-and-adults-2021-to-2026.pdf

[19] Reasons CE Hassan S Bige M Paras C Arora S. Race and criminal justice in Canada. Int. J. Criminal Justice Sci. (2016) 11:26. Available online at: https://digitalcommons.cwu.edu/cgi/viewcontent.cgi?article=1720&context=cotsfac

[20] Government of Canada. Understanding the Overrepresentation of Indigenous People in the Criminal Justice System. Available online at: https://www.justice.gc.ca/socjs-esjp/en/ind-aut/uo-cs

[21] Owusu-Bempah A. Race and policing in historical context: dehumanization and the policing of Black people in the 21st century. Theor Criminol. (2017) 21:23–34. 10.1177/1362480616677493

[22] Corrado RR Kuehn S Margaritescu I. Policy issues regarding the overrepresentation of incarcerated aboriginal young offenders in a Canadian context. Youth Justice. (2014) 14:40–62. 10.1177/1473225413520361

[23] Government of Canada. The Constitution Act, 1982. Schedule B to the Canada Act 1982. (1982). c 11. Available online at: https://www.canlii.org/en/ca/laws/stat/schedule-b-to-the-canada-act-1982-uk-1982-c-11/latest/schedule-b-to-the-canada-act-1982-uk-1982-c-11.html

[24] Cotter A. Experiences of discrimination among the Black and Indigenous populations in Canada, 2019. (2019) p. 14.30873095

[25] Armstrong A Jaffray B. Homicide in Canada, 2020. (2020). p. 36.

[26] Kingsley MF. Indigenous People in Federal Custody Surpasses 30% Correctional Investigator Issues Statement Challenge. Office of the Correctional Investigator (2020). Available online at: https://www.oci-bec.gc.ca/cnt/comm/press/press20200121-eng.aspx

[27] Salerno AC Schuller RA. A mixed-methods study of police experiences of adults with autism spectrum disorder in Canada. Int J Law Psychiatry. (2019) 64:18–25. 10.1016/j.ijlp.2019.01.002 31122628

[28] Tint A Palucka AM Bradley E Weiss JA Lunsky Y. Correlates of police involvement among adolescents and adults with autism spectrum disorder. J Autism Dev Disord. (2017) 47:2639–47. 10.1007/s10803-017-3182-5 28612245

[29] Copenhaver A Tewksbury R. Interactions between autistic individuals and law enforcement: a mixed-methods exploratory study. Am J Crim Justice. (2019) 44:309–33. 10.1007/s12103-018-9452-8

[30] Diamond LL Hogue LB. Preparing students with disabilities and police for successful interactions. Interv Sch Clin. (2021) 57:3–14. 10.1177/1053451221994804

[31] Government of Canada. Autism Spectrum Disorder Among Children and Youth in Canada 2018. (2018). Available online at: https://www.canada.ca/en/public-health/services/publications/diseases-conditions/autism-spectrum-disorder-children-youth-canada-2018.html

[32] Pietro N Illes J. Closing gaps: strength-based approaches to research with aboriginal children with neurodevelopmental disorders. Neuroethics. (2016) 9:1–10. 10.1007/s12152-016-9281-8

[33] Canadian Autism Spectrum Disorder Alliance. Working together to advance a National Autism Strategy. CASDA in the News. (2021). Available online at: https://www.casda.ca/working-together-to-advance-a-nas/

[34] *Canada PHA of*. Autism: National Autism Strategy. (2020). Available online at: https://www.canada.ca/en/public-health/services/diseases/autism-spectrum-disorder-asd/national-strategy.html

[35] CASDA, KBHN. Informing Canada's Autism Strategy: Lessons From Across the Globe. Canadian Autism Spectrum Disorder Alliance (2020). Available online at: https://www.casda.ca/wp-content/uploads/2020/07/CASDA-KBHN-InternationalStategies-June2020.pdf

[36] Government of Canada. Minister of Health Mandate Letter. Prime Minister of Canada (2021). Available online at: https://pm.gc.ca/en/mandate-letters/2021/12/16/minister-health-mandate-letter

[37] Truth and Reconciliation Commission of Canada. Calls to Action. (2015). Available online at: http://www.trc.ca/

[38] Committee on the Rights of Persons with Disabilities. Concluding Observations on the Initial Report of Canada. United Nations (2017). Available online at: http://docstore.ohchr.org/SelfServices/FilesHandler.ashx?enc=6QkG1d%2FPPRiCAqhKb7yhshFUYvCoX405cFaiGbrIbL87R7e4hNB%2FgZKnTAU8BqK7FKCyFSQGUzS4dKwSRSD%2FCPUoSzW7oP9OI5lweGr%2Br%2B7wpRzQbCN1rv%2B%2BwMd4F0fZ

[39] United Nations. Shared Framework on Leaving No One Behind: Equality Non-Discrimination at the Heart of Sustainable Development. Asia-Pacific Disaster Report 2015. New York, NY: UN (2016). Available online at: https://www.un-ilibrary.org/content/books/9789210576086c010

[40] Botha M Hanlon J Williams GL. Does language matter? Identity-first versus person-first language use in autism research: a response to vivanti. J Autism Dev Disord [Internet]. (2021). 10.1007/s10803-020-04858-w. [Epub ahead of print]. 33474662 PMC7817071

[41] Annamma SA Connor D Ferri B. Dis/ability critical race studies (DisCrit): theorizing at the intersections of race and dis/ability. Race Ethn Educ. (2013) 16:1–31. 10.1080/13613324.2012.730511

[42] Lane J. Black Peoples, Indigenous Peoples, People(s) of Colour (BIPOC): Inclusive Antiracist Writing. Simon Fraser University (2021). Available online at: https://www.lib.sfu.ca/about/branches-depts/slc/writing/inclusive-antiracist-writing/bipoc

[43] Deo ME. Why BIPOC Fails. Va Law Rev (2021). p. 107. Available online at: https://www.virginialawreview.org/articles/why-bipoc-fails/

[44] City of Ottawa. Equity and Inclusion Lens Snapshot - Racialized People. (2016). p. 20. Available online at: https://documents.ottawa.ca/sites/documents/files/racializd_ss_en.pdf

[45] Nicol J Osazuwa B. Race Ethnicity: Evolving Terminology. Library of Parliament. Social Affairs Population (2022). Available online at: https://hillnotes.ca/2022/01/31/race-and-ethnicity-evolving-terminology/

[46] Else-Quest NM Hyde JS. Intersectionality in Quantitative Psychological Research: I. Theoretical and epistemological issues. Psychol Women Q. (2016) 40:155–70. 10.1177/0361684316629797

[47] Namugenyi C Nimmagadda SL Reiners T. Design of a SWOT analysis model and its evaluation in diverse digital business ecosystem contexts. Procedia Comput Sci. (2019) 159:1145–54. 10.1016/j.procs.2019.09.283

[48] University of Alberta Library. Health Sciences Search Filters: Indigenous Peoples. (2022). Available online at: https://guides.library.ualberta.ca/health-sciences-search-filters/indigenous-peoples

[49] University of Prince Edward Island Robertson Library. Indigenous Peoples Search Terms. Available online at: https://library.upei.ca/indigenous-search-terms

[50] University of Toronto Libraries. Indigenous Health Resources: Databases. (2022). Available online at: https://guides.library.utoronto.ca/c.php?g=250466&p=5037464

[51] Page MJ McKenzie JE Bossuyt PM Boutron I Hoffmann TC Mulrow CD. The PRISMA 2020 statement: an updated guideline for reporting systematic reviews. BMJ. (2021) 372:n71. 10.1136/bmj.n71 33782057 PMC8005924

[52] Lindblom A. Exploring autism and music interventions through a First Nations lens. Altern Int J Indig Peoples. (2017) 13:202–9. 10.1177/1177180117729854

[53] Lindblom A. 'It gives them a place to be proud' - Music and social inclusion. Two diverse cases of young First Nations people diagnosed with autism in British Columbia, Canada. Psychol Music. (2017) 45:268–82. 10.1177/0305735616659553

[54] Gerlach A. Circle of caring: a first nations worldview of child rearing. Can J Occup Ther. (2008) 75:18–25. 10.1177/000841740807500107 18323364

[55] Simmons. Words have power: (re)-defining serious emotional disturbance for American Indian and Alaska native children and their families. Am Indian Alsk Native Ment Health Res. (2004) 11:59–64. 10.5820/aian.1102.2004.59 15322975

[56] Rossow-Kimball B Lavis M Blackhurst M. I can find my own Elder! Cultural engagement as serious leisure for Aboriginal adults living in non-Aboriginal group homes. Leis Stud. (2017) 36:244–55. 10.1080/02614367.2015.1085592

[57] Vargas T. I have to do what I have to do. The Washington Post. (2010). Available online at: https://web-s-ebscohost-com.libaccess.lib.mcmaster.ca/ehost/detail/detail?vid=0&sid=666bebf4-d25d-4796-8357-bb92cac77971%40redis&bdata=JnNpdGU9ZWhvc3QtbGl2ZSZzY29wZT1zaXRl#AN=WPT207297757210&db=bwh

[58] Carley M. Autism-Shmautism: in the end, Darius McCollum was Poor and Black. TCA EP World LLC. (2018) 48:34–8. Available online at: https://www.linkedin.com/pulse/autism-schmautism-end-darius-mccollum-poor-black-michael-john-carley

[59] Solomon O Lawlor MC. And I look down and he is gone: narrating autism, elopement and wandering in Los Angeles. Soc Sci Med. (2013) 94:106–14. 10.1016/j.socscimed.2013.06.034 23890970 PMC3788703

[60] Davenport MA Romero ME Lewis CD Lawson T Ferguson B Stichter J. An initial development and evaluation of a culturally responsive police interactions training for black adolescents with autism spectrum disorder. J Autism Dev Disord. (2021). 10.1007/s10803-021-05181-8. [Epub ahead of print]. 34448996

[61] Lisa Stevenson. Life Beside Itself: Imagining Care in the Canadian Arctic. University of California Press (2014). Available online at: https://www.ucpress.edu/book.php?isbn=9780520282940

[62] O'Gorman M. Mental and physical health impacts of water/sanitation infrastructure in First Nations communities in Canada: an analysis of the Regional Health Survey. World Dev. (2021) 145:105517. 10.1016/j.worlddev.2021.105517

[63] Inman CE. Absence and epidemic: autism and fetal alcohol spectrum disorder in indigenous populations in Canada. Can J Disabil Stud. (2019) 8:227–61. 10.15353/cjds.v8i4.531

